# Incidence and factors associated with central line-associated bloodstream infection in patients with chronic intestinal failure. A 20-year retrolective cohort

**DOI:** 10.1371/journal.pone.0340064

**Published:** 2026-01-06

**Authors:** Luis Eduardo González-Salazar, Pedro Antonio Verdeja-Padilla, Ana Luz Reyes-Ramírez, Adriana Flores-López, Lilia Castillo-Martínez, Arturo Galindo-Fraga, Alma Rosa Chávez-Ríos, Martha Asunción Huertas-Jiménez, Aurora Elizabeth Serralde-Zúñiga

**Affiliations:** 1 Servicio de Nutriología Clínica, Instituto Nacional de Ciencias Médicas y Nutrición Salvador Zubirán, Mexico City, Mexico; 2 Programa de Maestría y Doctorado en Ciencias Médicas, Odontológicas y de la Salud, UNAM, Mexico City, Mexico; 3 Subdirección de Epidemiología Hospitalaria y Control de la Calidad de la Atención Médica, Instituto Nacional de Ciencias Médicas y Nutrición Salvador Zubirán, Mexico City, Mexico; 4 Clínica de Catéteres Ambulatoria, Subdirección de Epidemiología Hospitalaria y Control de la Calidad de la Atención Médica, Instituto Nacional de Ciencias Médicas y Nutrición Salvador Zubirán, Mexico City, Mexico; 5 Subdirección de Enfermería, Instituto Nacional de Ciencias Médicas y Nutrición Salvador Zubirán, Mexico City, Mexico; Aga Khan University Hospital Nairobi, KENYA

## Abstract

**Background:**

Type 3 Intestinal Failure (IF-3) is a chronic condition in which patients may require long-term Outpatient Intravenous Supplementation (OIS) to maintain hydration and nutrition, they are susceptible to catheter-related infectious complications. The present study aimed to determine the incidence of factors associated with central line-associated bloodstream infection (CLABSI) in patients with IF-3 on OIS.

**Methodology:**

Retrolective cohort study of patients with IF-3 undergoing OIS treated at the Instituto Nacional de Ciencias Médicas y Nutrición Salvador Zubirán, a national tertiary care and referral center in Mexico City, between 2004 and 2024. Sociodemographic, anthropometric, biochemical, clinical, and microbiological data, including isolated pathogens, were collected. Variables associated with CLABSI incidence were determined using Poisson regression analysis.

**Results:**

Sixty patients were included, of which 31 (51.7%) were female and 29 (48.3%) were male. The mean age was 49.5 ± 18.1 years, and BMI was 19.8 ± 5.31 kg/m^2^. The primary pathophysiological mechanism of IF-3 was short bowel syndrome (n = 23 (38.3%)). Twenty-seven (45%) patients developed CLABSI, resulting in 36 events over 27,200 catheter days, with an incidence rate of 1.323 per 1,000 catheter days. Fifty-seven pathogens were isolated, the most frequent being Gram-negative bacteria (52.6%), specifically the *Enterobacter cloacae* complex (23.3%) and *Klebsiella oxytoca* (16.6%). Patients with intestinal stomas had a higher incidence of CLABSI (74% *vs* 48.4%; p = 0.039). Depression (IR 1.43; 95% CI 1.04–1.97, p = 0.028) was associated with a higher incidence of CLABSI.

**Conclusions:**

The incidence rate of CLABSI was lower than reported in other studies in upper-middle-income countries. Depression and the presence of intestinal stomas may be associated with a higher risk of CLABSI, possibly through their impact on self-care and stress-inflammation pathways. These results underscore the importance of psychosocial and clinical factors in preventing CLABSI. Multicentre studies are needed to enhance external validity across diverse settings.

## Introduction

Intestinal Failure (IF) is a limited condition that may be transient or irreversible and is caused by a reduced intestinal function below the minimum necessary to maintain absorption of macronutrients, water and/ or electrolytes, requiring intravenous supplementation to preserve individual health [1]. Type 3 IF (IF-3) is a chronic condition in which patients may require outpatient intravenous supplementation (OIS) of either fluids or parenteral nutrition through a central venous catheter (CVC) for months, years, or even a lifetime; only 20–50% achieve discontinuation after two years [1,2].

The global prevalence and incidence of IF-3 are uncertain. However, in Europe, a prevalence of 5–80 cases per million population and an annual incidence of 5–20 cases per million population have been reported [1,3]. In the United States, a prevalence of 75 cases per million population is estimated. In contrast, in middle-income countries such as Argentina, a prevalence of 0.25–6.75 per million population has been reported [3,4], probably due to underreporting. Most of the literature refers to the high economic costs of OIS, but this cost is 60–70% lower compared to hospital parenteral nutrition [5].

The management of IF-3 is a multidisciplinary approach to achieve adequate treatment of the underlying gastrointestinal disease, optimal delivery of OIS, and prevention of complications related to long-term use, such as catheter-related bloodstream infections (CRBSI). CRBSI is used to describe bloodstream infections, whether for central or peripheral lines; the definition is based on laboratory testing that involves isolating the microorganism from both blood drawn from the catheter lumen and the peripheral vein of a patient with clinical symptoms of infection [6–8]. Whereas central line-associated bloodstream infection (CLABSI) is a term more commonly used for surveillance purposes and, as its name suggests, refers specifically to infections associated with central lines, it employs the same methodology as CRBSI to diagnose it [6,8–10].

CLABSI are the most frequent and severe OIS-related complications, increasing morbidity and mortality in patients with IF-3 [2,11,12]. According to the International Consortium for Nosocomial Infection Control report, CLABSI is associated with a crude mortality of 24.9%, a mean in-hospital stay of 19.47 days, and an estimated USD 2,619.00 hospital cost [13].

In patients receiving OIS, specifically with home parenteral nutrition (HPN), an incidence rate of CLABSI of 1.65 (95% CI 1.09–2.84) events per 1000 days/catheter has been reported [14]. In comparison, the incidence rate of CRBSI is 0.38–4.58 episodes per 1000 days/ catheter [6]. It has been reported that the most common organisms that cause CLABSI or CRBSI are Gram-positive bacteria (53%), followed by Gram-negative bacteria (26%) and yeast (12%) [14].

The leading causes of CLABSI are contamination of the catheter connector and migration of skin organisms at the catheter insertion site. Several risk factors for CLABSI have been described in the literature that may be related to patient and CVC device characteristics. Patient-related risk factors include the type of underlying disease, use of immunosuppressants, thrombosis, age, and method of parenteral nutrition infusion. On the other hand, risk factors associated with CVC include catheter gauge (>2 mm), number of lumens, and the type of cannulated vein, among others [6,15,16]. In addition, in patients receiving OIS, there is a complex bidirectional relationship between catheter infections and psychosocial factors, particularly depression. Infections are a major source of stress and emotional trauma contributing to depression and may also influence CLABSI risk, as depressive symptoms are common in patients requiring long-term OIS and may impair adherence to catheter-care protocols and other self-management behaviors, affect the prevention of future infections [17].

However, most of the risk factors described have been obtained from studies conducted in countries with high- or middle-income levels or specialized units in managing patients with IF. For this reason, this study aims to determine the incidence and factors associated with CLABSI in patients with IF-3 and OIS at the Instituto Nacional de Ciencias Médicas y Nutrición Salvador Zubirán (INCMNSZ), a national tertiary care and referral center in Mexico City.

## Methodology

### Study design

A retrolective cohort study was conducted at the INCMNSZ, a national tertiary care and referral center in Mexico City, which provides specialized care to adult patients with complex gastrointestinal disorders from across the country. INCMNSZ is the only public hospital in Mexico that provides follow-up care for patients with IF-3. Adult patients who had initiated OIS between January 2004 and April 2024 were included, resulting in 70 possible patients to be analyzed. Patients were excluded if they had persistent infections, antimicrobial therapies initiated within the first 14 days after OIS hospital discharge, or incomplete information in their clinical record or failed to follow up. This study was conducted following the guidelines of the Declaration of Helsinki, and all human subject procedures were approved by the INCMNSZ Ethics Research Committee (REF 2119) (consent was not obtained because the data were analyzed anonymously). No patients contact occurred and no reimbursement or compensation was provided.

### Dependent variable

**Fig 1:**
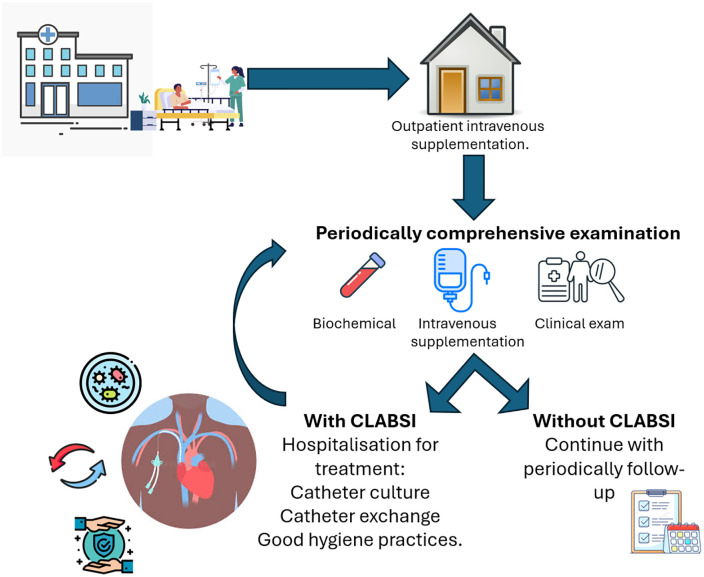
Outpatient intravenous supplementation program. Hospitalized patients who can continue receiving Outpatient Intravenous Supplementation undergo training in the correct manipulation of the catheter to avoid infections. These patients have periodic examinations to ensure the safety of the catheter and the adequacy of supplementation, among other parameters. CLABSI: Central Line-Associated Bloodstream Infection.

The dependent variable was the incidence of CLABSI, reported as events per 1000 days/catheter. Patients were diagnosed with CLABSI if they had evidence of bloodstream infection confirmed by blood cultures and positive CVC cultures in the absence of another primary site of infection [6,9]. In accordance with the Centers for Disease Control and Prevention (CDC)/National Healthcare Safety Network (NHSN) criteria, a CLABSI was considered only when the patient had a central line in place for more than two consecutive days before the date of the bloodstream infection event. For organisms considered common commensal, the diagnosis required at least two positive blood cultures obtained on the same day but drawn from separate venipunctures, as specified by NHSN laboratory-confirmed bloodstream infection criteria [18]. The diagnosis was established by the specialized medical team in collaboration with the hospital epidemiology service, following standardized institutional criteria. Patients identified with CLABSI are hospitalized to undergo catheter culture, exchange and to reinforce good hygiene practices (Fig 1).

### Independent variables

Independent variables included demographic characteristics (sex and age), clinical factors (comorbidities, pathophysiological mechanism and underlying etiology of intestinal failure), anthropometric parameters (body weight and body mass index (BMI)), presence of enteral access, type and days of OIS, type of CVC (standard, Hickman, or implanted port), catheter characteristics (site of insertion, tunneling, type and frequency of infusions, and additional catheter use), biochemical and microbiological parameters.

### OIS program description

OIS was defined as the presence of outpatient administration of intravenous fluids and/or parenteral nutrition through the CVC. Polymicrobial infection was defined as the presence of more than one pathogen in the culture isolation. The IF-3 patients OIS program has a multidisciplinary professional team that specializes in nutritional support, including physicians, dieticians, and nurses, who provide training and education on catheter care to patients and their family members.

In addition, nutritional, clinical, and metabolic monitoring is performed every 7–14 days to ensure proper management of OIS and to prevent or detect possible complications in a timely manner.

Throughout the 20 years of the OIS program, several infection control measures were progressively implemented. Initially, catheter site care involved the use of 70% alcohol and 10% povidone-iodine. In 2012, these were replaced with a solution containing 70% alcohol and 2% chlorhexidine. That same year, chlorhexidine-based cleaning (0.12% and 2%) was introduced before catheter placement, and patients were instructed to perform modified bathing at home to reduce moisture near the catheter dressing. Psychological and psychiatric support was also recommended as part of a comprehensive care strategy to promote adherence and infection prevention [9,10,16].

### Data extraction

Data collection was performed using a standardized form specifically designed for this study. To minimize the risk of bias, all data were extracted from the clinical record by a single trained researcher, such as demographic, biochemical, clinical (comorbidities, pathophysiology and etiology of IF), nutritional, anthropometric variables, presence of enteral access, days with OIS, type of OIS (fluids and/or parenteral nutrition), type of device, characteristics of CVC, termination of OIS, additional use of the catheter, those related to CLABSI (characteristics of the isolated microorganisms) and mortality. Data extraction from the clinical records was conducted between January 6^th^ 2023 and October 31^th^ 2024. Two independent researchers reviewed the data to ensure consistency and accuracy. Researchers did not have access to information that could identify individual participants.

### Biochemical data

Biochemical parameters were obtained at the beginning of OIS, complete blood count (CBC) test (hemoglobin, hematocrit, leukocytes, platelets), comprehensive metabolic panel (total, direct and indirect bilirubin, alanine aminotransferase (ALT), aspartate aminotransferase (AST), alkaline phosphatase, albumin, glucose, creatinine, blood urea nitrogen (BUN)), electrolyte panel (sodium, potassium, chloride, calcium, magnesium, phosphorus), C reactive protein (CRP) and triglycerides.

### Clinical covariates

The history of use of alcohol and tobacco before or during the use of OIS was obtained from clinical records. Comorbidities were obtained as well from clinical records before or during the use of OIS (chronic or acute kidney disease, heart failure, intestinal- failure associated liver disease (IFALD), sepsis, pneumonia, thrombosis and osteoporosis, type 2 diabetes (T2D), high blood pressure, and depression (defined as the presence of a depressive-anxiety disorder diagnosed by a medical specialist). Missing data were handled on a case-by-case basis. When information was not available in the medical records, it was recorded as “not reported” and excluded from specific statistical analyses. No imputation methods were used. Patients were followed from enrollment in the OIS program until the last documented follow-up or the end of the study period. Patients with insufficient follow-up information were excluded from the analysis.

This study did not include a control group, as its primary objective was to describe the incidence of CLABSI and identify associated factors within a defined cohort of patients with IF-3 with OIS.

### Statistical analysis

All categorical variables were expressed as frequency (percentage), and continuous variables were expressed as mean (standard deviation (SD)) if they were normally distributed or median (interquartile range, IQR) if they were non-normally distributed. Comparison between categorical variables was performed using the Pearson’s χ2 test or Fisher’s exact test. An independent T-test or Mann-Whitney test was used to measure continuous variables.

The incidence of CLABSI was reported as an event per 1000 days/catheter. Poisson regression analysis was performed to identify potential factors associated with the incidence rate (IR) of CLABSI, considering confidence intervals (95% CI), and model performance was evaluated using the Akaike Information Criteria (AIC) and the ratio of deviance; p < 0.05 was considered statistically significant. Additionally, we performed a multivariate Poisson regression analysis, including potential confounders as age, sex, and BMI. Statistical analysis was performed using SPSS software (Windows, version 24, SPSS IBM Inc.), and figures were created in the GraphPad Prism software (Windows, version 8.0). S1 File presents the final preprocessed dataset used in this study.

## Results

### Characteristics of the study population

**Fig 2:**
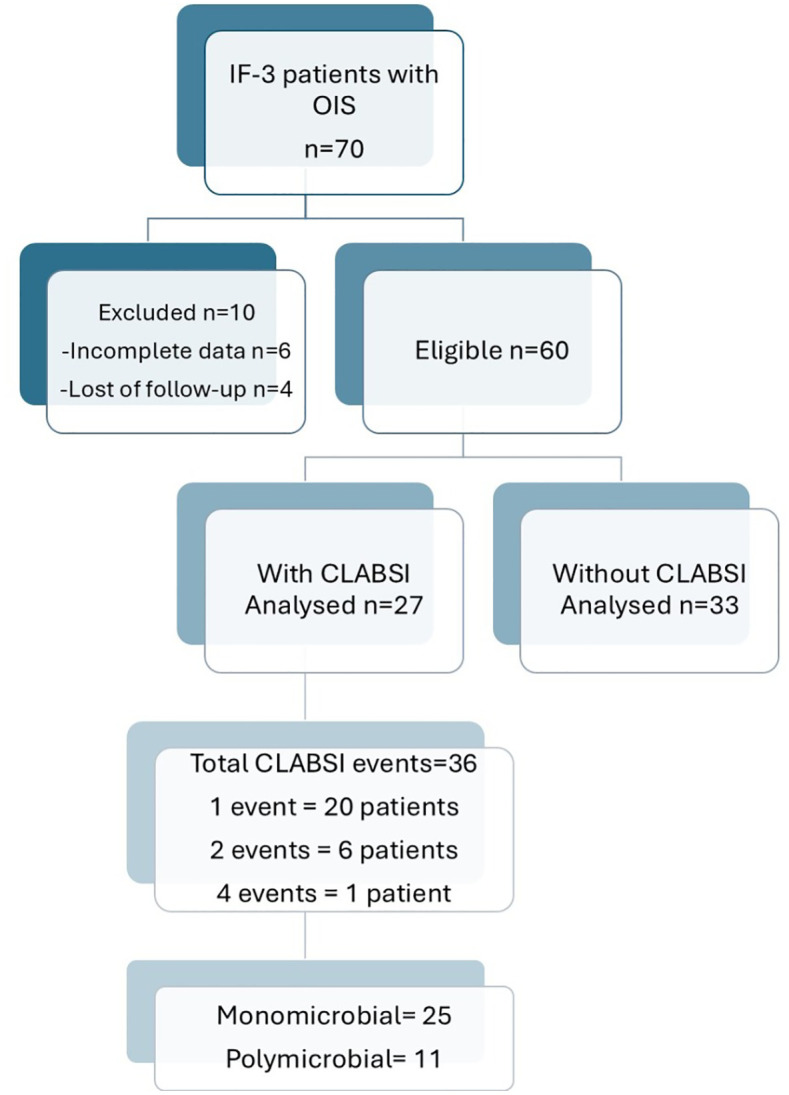
Flowchart diagram. IF-3: type 3 Intestinal Failure; OIS: Outpatient Intravenous; CLABSI: Central Line-Associated Bloodstream Infection.

Seventy patients with IF-3 and OIS were eligible, of which 60 were included (51.7% female, 48.3% male), with a mean age of 49.5 ± 18.1 years and body mass index of 19.8 ± 5.31 kg/m^2^. Of the excluded patients, 6 had incomplete data on file, and four were lost to follow-up (Fig 2).

**Fig 3:**
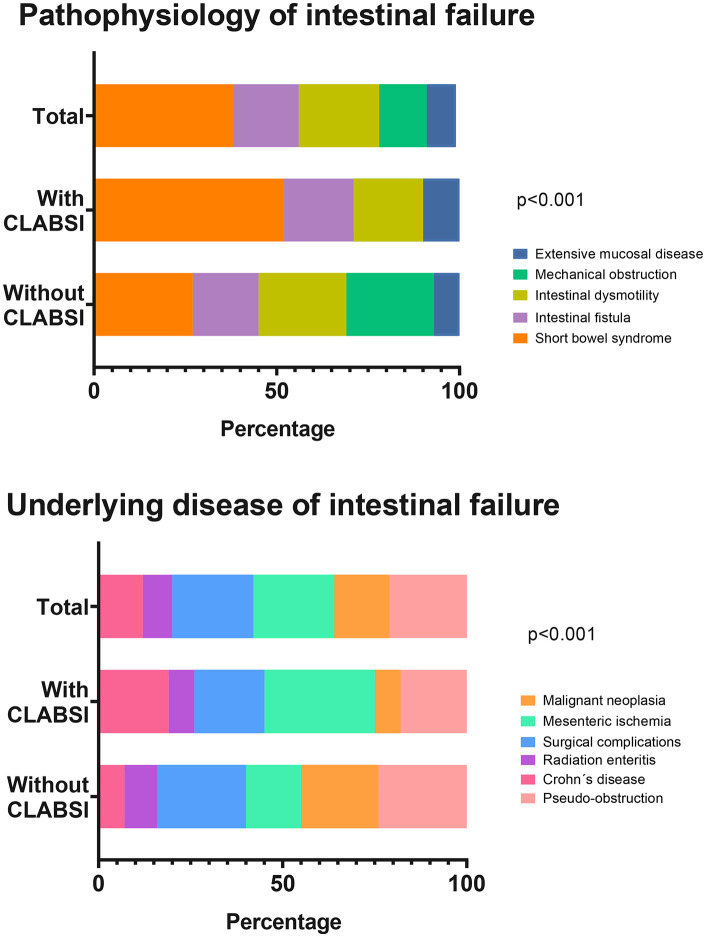
Pathophysiology and underlying disease of intestinal failure in total cohort and patients with and without Central Line-Associated Bloodstream Infection (CLABSI). Data are presented as percentages. Global chi-square or Fisher´s exact test analysis was performed according to the expected frequencies. A p-value < 0.05 was considered statistically significant.

The most frequent pathophysiological mechanisms of IF-3 were short bowel syndrome (38.3%), intestinal dysmotility (21.6%), and intestinal fistulas (18.3%). Mesenteric ischemia, surgical complications, intestinal pseudo-obstruction, and malignant neoplasms were the most frequent etiological causes of IF-3 with 15% (Fig 3).

The main comorbidities present were depression (58.3%; n = 35), acute kidney injury (46.7%; n = 28), osteoporosis (36.6%; n = 22), thrombosis (30%; n = 18), and smoking history (40%; n = 40). The median time of OIS was 267.5 (IQR: 130.2–570.7) days and 27,200 catheter days, 48.3% (n = 29) of patients had a standard 3-lumen polyurethane catheter (Arrow®) as their first central venous access device, and 86.6% (n = 52) of catheters were tunnelled. The 18% (n = 11) of patients used central venous access exclusively for fluid and/or electrolyte, 20% (n = 12) exclusively for parenteral nutrition, and 62% (n = 37) received both; 28.3% (n = 17) additionally used the CVC to administer solutions other than OIS (Table 1).

**Table 1:** Demographic characteristics of the study population.

Characteristics	Total N = 60	Patients without CLABSI N = 33	Patients with CLABSI N = 27	p-value
Age, mean years (SD)	49.5 (18.1)	53 (16.5)	45.2 (19.4)	0.10
Weight, mean Kg (SD)	52.2 (15.6)	53.5 (16.5)	50 (14.5)	0.47
BMI, mean Kg/m^2^ (SD)	19.8 (5.31)	20.5 (6.05)	18.9 (4.16)	0.24
Sex, frequency (%)				
Male Female	29 (48.3) 31 (51.7)	15 (45.5) 18 (54.5)	14 (51.8) 13 (48.2)	0.81
Pathophysiology of intestinal failure, frequency (%)				
Short bowel syndrome Intestinal fistula Intestinal dysmotility Mechanical obstruction ESBMD	23 (38.3) 11 (18.3) 13 (21.6) 8 (13.3) 5 (8.3)	9 (27.2) 6 (18.1) 8 (24.2) 8 (24.2) 2 (6.0)	14 (51.8) 5 (18.5) 5 (18.5) 0 3 (11.1)	**0.051** 0.97 0.59 **0.006** 0.48
Underlying disease, frequency (%)				
Crohn´s disease Radiation enteritis Surgical complications Mesenteric ischemia Malignant neoplasia Pseudo-obstruction	7 (11.6) 5 (8.3) 13 (21.6) 13 (21.6) 9 (15) 13 (21.6)	2 (6.1) 3 (9.0) 8 (24.3) 5 (15.1) 7 (21.2) 8 (24.3)	5 (18.5) 2 (7.4) 5 (18.5) 8 (29.6) 2 (7.4) 5 (18.5)	0.13 0.81 0.59 0.17 0.13 0.59
Type central venous access, frequency (%)				
Standard (Arrow) Hickman Implanted port	29 (48.3) 20 (36.4) 11 (28.3)	14 (42.4) 12 (36.3) 7 (21.2)	15 (55.5) 8 (29.6) 4 (14.8)	0.31 0.58 0.52
Duration of OIS	267.5 (130.2-570.7)	229 (102-441.5)	419 (164-576)	0.18
Tunneling				
Tunneled	52 (86.6)	28 (84.8)	24 (88.8)	0.64
Site of insertion				
Right Jugular Left Jugular Right Subclavian Left Subclavian	46 (76.6) 3 (5.0) 10 (16.6) 1 (1.6)	29 (87.8) 1 (3.0) 3 (9.0) 0	17 (62.9) 2 (7.4) 7 (25.9) 1 (3.7)	**0.023** 0.43 0.08 0.45
Type of infusion				
Hydration Parenteral Nutrition Both	11 (18) 12 (20) 37 (62)	6 (18.2) 9 (27.3) 18 (54.5)	5 (18.5) 3 (11.1) 19 (70.4)	0.97 0.19 0.28
Frequency of infusions				
Daily	43 (71.6)	24 (72.7)	19 (70.3)	0.84
Additional use	17 (28.3)	8 (24.2)	9 (33.3)	0.43
Other devices, frequency (%)				
Enteral/oral nutrition Stoma Decompressive gastrostomy	52 (86.6) 36 (60) 11 (18.3)	27 (81.8) 16 (48.4) 6 (22.2)	25 (92.5) 20 (74) 5 (18.5)	0.22 **0.044** 0.97
Comorbidities, frequency (%)				
Tobacco Alcoholism Type 2 diabetes Hypertension Chronic kidney disease Heart failure Acute kidney injury Depression NALD Sepsis Pneumonia Thrombosis Osteoporosis	24 (40) 11 (18.3) 6 (10) 7 (11.6) 10 (16) 13 (21.6) 28 (46.7) 35 (58.3) 15 (25) 23 (38.3) 17 (28.3) 18 (30) 22 (36.6)	16 (48.4) 6 (18.1) 1 (3.0) 3 (9.0) 4 (12.1) 5 (15.1) 14 (42.4) 14 (42.4) 8 (24.2) 11 (33.3) 8 (24.2) 9 (27.2) 13 (39.3)	8 (29.6) 5 (18.5) 5 (18.5) 4 (14.8) 6 (22.2) 8 (29.6) 14 (51.8) 21 (77.7) 7 (25.9) 12 (44.4) 9 (33.3) 9 (33.3) 9 (33.3)	0.13 0.97 **0.047** 0.49 0.29 0.17 0.46 **0.008** 0.99 0.37 0.43 0.61 0.62
Mortality, frequency (%)	13 (21.6)	6 (18.1)	7 (25.9)	0.53

ESBMD: Extensive small bowel mucosal disease. PNALD: Parenteral nutrition associated liver disease. OIS: outpatient intravenous supplementation. CLABSI: Central Line Associated Bloodstream Infections.

Continuous variables are expressed as mean (SD) or median (interquartile range, IQR). Qualitative variables are presented as frequency (%). Statistical analysis was performed using the independent t-test or Mann-Whitney test for continuous variables. Statistical analysis of qualitative variables was performed with the χ2 test. A p-value < 0.05 was considered statistically significant.

### Incidence and clinical characteristics of patients with CLABSI

We documented that 27 (45%) patients developed CLABSI; 7 patients (26%) developed more than one CLABSI event, and in total, 36 events were analyzed, resulting in an incidence rate of 1.323 events per 1000 days/central venous catheter. Of the patients who did not have CLABSI, 10 (30.3%) discontinued OIS due to recovery of bowel function, 11 (33.3%) had transferred to another healthcare institution, and 12 (36.3%) patients continued OIS during follow-up.

There was a higher proportion of patients with a history of T2D (18.5% vs 3.0%; p = 0.047) and depression (77.7% vs 42.4%; p = 0.008) in the CLABSI group (Table 1). In the group of patients with CLABSI, a trend toward a higher proportion of patients with short bowel syndrome (51.8% vs 27.2%; p = 0.051) and a significantly lower proportion of mechanical obstruction (0% vs 24.2%; p = 0.006) were observed. Furthermore, a significantly higher presence of intestinal stomas was observed in CLABSI patients (74% vs. 48.4%; p = 0.044).

Interestingly, CLABSI patients had significantly less CVC insertion in the right jugular vein (62.9% *vs* 87.8%; p = 0.023), and there was a trend towards greater use of the right subclavian vein (25.9% *vs* 9.0%; p = 0.08) compared to the group of patients without CLABSI (Table 1).

Additionally, of the total CLABSI events (n = 36), 52.8% occurred in patients with a standard (Arrow) catheter, 25% with a Hickman catheter, and 22.2% with an implanted port. Most CLABSI episodes involved tunneled catheters (77.7%). Regarding insertion site, the right internal jugular vein was the most frequent (61.1%) (Table 2).

**Table 2:** Catheter related characteristics among central line-associated bloodstream infection events.

Catheter characteristics	n = 36
**Type central venous catheter, frequency (%)**	
Standard (Arrow)	19 (52.8)
Hickman	9 (25)
Implanted port	8 (22.2)
**Tunneling, frequency (%)**	
Tunneled	28 (77.7)
**Site of insertion, frequency (%)**	
Right Jugular	22 (61.1)
Left Jugular	4 (11.1)
Right Subclavian	8 (22.2)
Left Subclavian	2 (5.6)

No significant difference was observed in metabolic and other biochemical parameters (S1 Table). Of the group of patients who had CLABSI, seven patients (25.9%) died during the follow-up; two died due to septic shock, two due to pneumonia, two from cholangitis complications, and one due to COVID-19. The time elapsed between the CLABSI event and death was a mean of 340 ± 120 days.

### Factors related to CLABSI

In a univariate analysis, a significantly higher incidence of CLABSI was observed in patients with a history of depression (IR 1.43; 95% CI 1.04–1.97; p = 0.028) (S2 Table). In the multivariate Poisson regression model, depression showed a positive trend toward a higher incidence of CLABSI (incidence rate (IR) 2.38; 95% CI: 0.93–6.04), although this was not statistically significant (p = 0.06) (S3 Table). Smoking also showed a trend toward association with polymicrobial CLABSI (IR 2.85; 95% CI 0.95–8.48, p = 0.06) (S4 Table).

### Pathogen characteristics

Of the total CLABSI events (n = 36), 30.5% (n = 11) were polymicrobial and 69.4% (n = 25) were monomicrobial. From the 36 CLABSI events observed in 27 patients, a total of 57 pathogens were isolated. Of these, 30 (52.6%) were Gram-negative bacteria, 18 (31.6%) Gram-positive bacteria, and 9 (15.8%) fungi. A higher proportion of *Enterobacter cloacae* complex species (n = 7; 23.3%) and *Klebsiella oxytoca* (n = 5; 16.6%) were identified within the Gram-negative bacteria.

**Fig 4:**
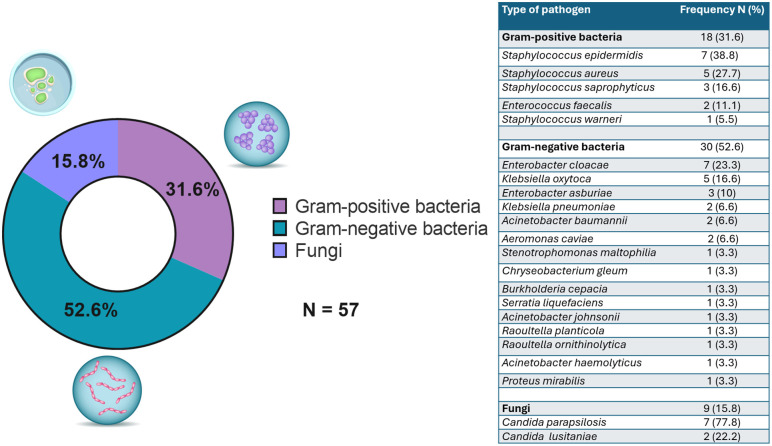
Proportion of total pathogens causing Central Line-Associated Bloodstream Infection.

Among the Gram-positive bacteria, a higher proportion of *Staphylococcus epidermidis* (n = 7; 38.8%), *Staphylococcus aureus* (n = 5; 27.7%), and *Staphylococcus saprophyticus* (n = 3; 16.6%) was observed, while among the fungi, a higher proportion of *Candida parapsilosis* (n = 7; 77.8%) was observed (Fig 4). Of the seven patients with recurrent infections, four (57%) were caused by Gram-negative bacteria, and 3 (43%) were caused by Gram-positive bacteria.

## Discussion

This study describes the incidence, risk factors, and microbiological profile of CLABSI in patients with IF-3 and OIS in Mexico. We found that 45% of patients developed CLABSI, with an incidence of 1.323 episodes per 1000 catheter days, based on data collected over a 20-year period (January 2004-April 2024). Most isolated pathogens were Gram-negative bacteria, followed by Gram-positive bacteria and fungi.

We observed significant associations between CLABSI and clinical variables such as depression, T2D, smoking, and intestinal stomas. As a national tertiary referral center, our patients frequently present with multiple comorbidities such as depression, thrombosis, and acute kidney injury, which may themselves contribute to the increased risk of CLABSI rather than being consequences of CLABSI therapy. In addition, some of these comorbidities are common in patients with IF-3 due to the long-term complications of parenteral nutrition [19].

Few studies in Latin America evaluate the incidence and factors associated with CLABSI in patients with IF-3 and OIS. Most studies reporting the incidence and risk factors related to CLABSI in the OIS population have been conducted in countries with a specialized center for IF, such as those in Europe or the United States. A systematic review conducted in 2019 reported a summary random-effects rate of CLABSI of 1.65 (95% CI: 1.09–2.48) episodes per 1000 catheter days in patients with HPN. In this study, 45% of patients developed CLABSI with an incidence rate of 1.323 episodes per 1,000 catheter days, lower than reported in the systematic review [14].

This difference in incidence could be the reflection of the long-standing structure of our OIS program, which includes standardized catheter-care education, chlorhexidine-based protocols, and close outpatient monitoring every 7–14 days. These protocols have also evolved over the 20-year study period, incorporating progressive improvements in antisepsis, patient training, and psychosocial support.

In Latin America, we identify only one prospective study in Argentina involving 56 patients with IF-3 due to small bowel syndrome, in which a CLABSI prevalence of 25% over a 3-year follow-up period was reported; nonetheless, the incidence rate was not reported [4]. In Brazil, in patients with short bowel syndrome receiving HPN, an incidence of CLABSI of 0.88 episodes per patient-year over a 7-year follow-up period has been reported [20]. Meanwhile, a multicenter study involving five Latin American countries (Mexico, Argentina, Colombia, Chile, and Uruguay) reported a CLABSI incidence rate of 2.58 episodes per 1,000 catheter days; however, only patients in intensive care units were included [21].

On the other hand, we found a higher proportion of subjects with a history of T2D in the group that developed CLABSI. Previous studies have associated this pathology as an independent risk factor for CRBSI in intensive care units, given that this condition may result in a lower immune response [22]. T2D may predispose to CLABSI through chronic hyperglycemia, which impairs neutrophil chemotaxis, phagocytosis, and macrophage activation, leading to deficient innate immunity [23,24].

In the regression analysis, depression was associated with a significant incidence of CLABSI. This variable may be important to assess in OIS patients, as it has been reported that up to 80% of patients receiving HPN have depression symptoms, which affects the quality of life (QoL) and self-care of the CVC [25]. Moreover, HPN itself has been associated with a substantial impact on QoL, including psychosocial burden, restrictions in daily and social activities, and increased emotional distress.

These challenges may contribute to or exacerbate depressive symptoms, further compromising self-care and increasing the risk of catheter-related complications [25,26]. On the other hand, biological mechanisms may also contribute, as depression has been linked to stress–inflammation pathways involving increased activation of pro-inflammatory cytokines and dysregulation of the hypothalamic–pituitary–adrenal axis, which could increase susceptibility to infections [27].

These emphasize the need to integrate psychosocial screening and targeted education within OIS programs. Incorporating routine mental health screening as part of this approach may help identify patients at greater risk and allow timely support to strengthen catheter-care adherence. This is particularly relevant because depressive symptoms can reduce adherence to catheter-care practices and impair self-management in the home setting [7]. Additionally, we observed a trend toward higher CLABSI incidence in patients with more than six comorbidities (p = 0.08). This is consistent with reports showing that a greater comorbidity burden is associated with increased risk of CLABSI and worse clinical outcomes [28,29].

We also found that smoking may be a factor associated with the incidence of polymicrobial infection in patients with CLABSI (p = 0.06). Previous studies have described an association between smoking and a decreased immune system and increased colonization of bacteria in the catheter tip [30,31]. An extensive prospective cohort study reported that current smokers had a 41% higher risk of developing bloodstream infection (HR: 1.41; 95% CI 1.22–1.63) compared to never-smokers [32].

In the CLABSI group, the presence of stoma was higher than in the group without CLABSI; their presence is related to the patient’s underlying diagnosis (short bowel syndrome, motility disorders) and may serve as a direct communication route between bacteria from the gastrointestinal tract and the skin. Intestinal fluid output contains a variety of microorganisms, which could potentially increase the risk of catheter exposure [33,34].

We documented that of the total pathogens isolated, there was a modest majority were Gram-negative bacteria, followed by Gram-positive bacteria and fungi. This is a notable finding that differs from global trends, where Gram-positive bacteria are typically predominant in HPN cohorts. Dreesden *et al*., reported in a systematic review of 39 articles that 61% of the pathogens were Gram-positive, 23% were Gram-negative, and 8% were fungi [6]. Meanwhile, Reitzel *et al*. reported a similar prevalence of Gram-positive bacteria, at 53% [14]. This difference can be partly explained by the type of patients included in our study, where we observed that 60% of our population had stomas, which is associated with a higher presence of Gram-negative bacteria.

According to Stoma I. *et al.*, the intestinal predominance of Gram-negative bacteria was considered a predictor of CRBSI, which was observed globally and individually at the genus level: *Escherichia, Klebsiella, Enterobacter, Pseudomonas,* and *Stenotrophomonas* [35]. Although several of these pathogens are typically described as hospital-associated microorganisms, in our cohort, they most likely reflect intestinal colonization and community or household exposure, particularly in patients with stomas. *Enterobacter cloacae* complex has been reported as part of the human gut microbiota and can precede invasive infections [36,37], while *Pseudomonas aeruginosa* has also been identified in community carriers outside the hospital environment [38].

Furthermore, catheter-related bloodstream infections caused by *K. oxytoca* have been previously documented, particularly in immunocompromised or device-dependent populations such as oncology and chemotherapy patients, as well as in hospital settings where contaminated multidose vials or prolonged catheter use have facilitated transmission [39]. This suggests that, in IF-3 patients, the predominance of Gram-negative bacteria may derive from both endogenous and community reservoirs, underscoring the need for strict catheter-care education in the outpatient setting.

The catheter insertion site affects the risk for catheter related infection and phlebitis. The risk for catheter infection in part can be related to the risk for thrombophlebitis (right-sided access should be preferred to the left-sided approach to reduce the risk of thrombosis) and the density of local skin flora. Proximity to wounds, prior exit sites, tracheotomies, stomas or fistulae should be avoided. Femoral catheters are associated with a higher risk of infection and deep venous thrombosis, than internal jugular or subclavian catheters and should also be avoided, where possible [40].

In addition, we observed that patients with CLABSI had a lower utilization of the right jugular vein and a trend of higher utilization of the right subclavian vein for CVC insertion. The site of CVC insertion is still controversial, with some guidelines recommending the use of the right jugular vein compared to peripherally inserted CVCs (PICC) and femoral insertion sites; this has a lower risk of thrombosis and catheter-associated complications [7].

These findings may also reflect practical differences in catheter maintenance, the right internal jugular vein often provides better visualization and more stable placement in ambulatory settings, which may help reduce accidental manipulation and contamination [41]. On the other hand, the type of CVC used was not associated with CLABSI development in our cohort. A recent meta-analysis reported higher CLABSI rates in multi-lumen catheters compared with single-lumen devices [42]; however, our sample size may have limited the ability to detect similar patterns in this cohort.

Although studies report the incidence of CLABSI, there has been no evaluation of associated factors in a cohort of patients with chronic IF in Latin America, which is a strength. Nevertheless, our study has limitations due to its retrospective design and the relatively small sample size of the study, which is particularly relevant for subgroup analyses. These factors may have contributed to the presence of borderline p-values, such as the association with short bowel syndrome (p = 0.051) and the use of the right subclavian vein (p = 0.08). This limitation could also explain why depression, although significant in the univariate analysis, did not remain statistically significant in the multivariate analysis, likely reflecting limited sample size and statistical power.

In this context, a larger sample size, prospectively and with a more in-depth exploration of patients with OIS-dependent IF-3, could generate relevant information. Finally, the generalizability of our findings to other hospitals may be limited, as the INCMNSZ is a national referral center with complex population that does not reflect those of general hospitals or community-based OIS program. Nevertheless, this study provides valuable insights into the experience and effectiveness of an OIS program serving as a starting point for future research. These findings may guide preventive strategies in the OIS program, while future multicenter studies are needed to determine whether this pattern is consistent in other settings.

## Conclusion

Our findings show that IF-3 patients on OIS remain with a higher risk of developing CLABSI, with an incidence of 1.32 per 1000 catheter-days. Depression and intestinal stomas were associated with higher risk, and a modest predominance of Gram-negative bacteria. As this is a single-center study in Mexico, generalizability is limited; multicenter studies are needed to enhance external validity across diverse settings. However, these findings reinforce the need to align mental health assessment with structured infection-prevention strategies as part of OIS care. For clinicians, adopting individualized risk stratification and closer follow-up may help anticipate complications in IF-3 patients. In addition, establishing standardized protocols for culture reporting and longitudinal resistance tracking could enhance surveillance efforts and support more effective CLABSI-prevention strategies in future cohorts.

## Supporting information

S1 File Database used in this study. (XLSX)

S1 Table Biochemical characteristics of the study population. (DOCX)

S2 Table Poisson regression analysis of variables associated with the incidence of CLABSI. (DOCX)

S3 Table Multivariate Poisson regression analysis of variables associated with the incidence of CLABSI. (DOCX)

S4 Table Poisson regression analysis of variables associated with the incidence of polymicrobial CLABSI. (DOCX)
