# Supplementary material for: Incidence and factors associated with central line-associated bloodstream infection in patients with chronic intestinal failure. A 20-year retrolective cohort
Source: PLoS One. 2026 Jan 6;21(1):e0340064. doi: 10.1371/journal.pone.0340064 (PMC12774362; doi:10.1371/journal.pone.0340064)
Supplement: S1 Table — (DOCX) [file pone.0340064.s002.docx]

**Table S2. Biochemical characteristics of the study population.**

| Characteristics | Total  N= 60 | Patients without CLABSI  N= 33 | Patients with CLABSI  N= 27 | P-value |
| --- | --- | --- | --- | --- |
| Hemoglobin, g/dL | 10.8 (9.30, 12.2) | 10.5 (9.05, 12.5) | 11.0 (9.5, 12.0) | 0.57 |
| Hematocrit, % | 32.8 (28.3, 36.7) | 32.5 (28.0, 38.8) | 32.8 (28.8, 36.2) | 0.78 |
| Leukocytes, x 10^3/μL | 5.20 (3.80, 7.35) | 5.10 (3.95, 7.43) | 5.60 (3.75, 7.40) | 0.84 |
| Neutrophils, % | 66.5 (56.3, 73.2) | 69.6 (57.6, 76.3) | 63.5 (52.5, 72.5) | 0.39 |
| Platelets, x 10^3/μL | 270 (197, 341) | 280 (199, 362) | 242 (179, 317) | 0.18 |
| Total bilirubin, mg/dL | 0.53 (0.32, 0.96) | 0.56 (0.30, 0.89) | 0.50 (0.34, 1.03) | 0.75 |
| Direct bilirubin, mg/dL | 0.18 (0.10, 0.35) | 0.19 (0.10, 0.32) | 0.16 (0.10, 0.51) | 0.96 |
| Indirect bilirubin, mg/dL | 0.35 (0.23, 0.58) | 0.33 (0.22, 0.57) | 0.38 (0.23, 0.66) | 0.77 |
| ALT, U/L | 31.5 (17.9, 52.5) | 33.0 (18.0, 51.3) | 29.8 (17.2, 55.4) | 0.86 |
| AST, U/L | 27.5 (19.6, 40.5) | 28.0 (18.0, 40.0) | 26.8 (20.0, 51.2) | 0.72 |
| Alkaline phosphatase, U/L | 140 (87.0, 232) | 117 (82.0, 226) | 152 (109, 270) | 0.12 |
| Albumin, g/dL | 3.23 (2.80, 3.54) | 3.25 (2.70, 3.62) | 3.21 (2.99, 3.50) | 0.77 |
| Glucose, mg/dL | 95.0 (84.5, 103) | 94.5 (82.7, 101.2) | 96.0 (84.5, 104) | 0.54 |
| BUN, mg/dL | 17.4 (12.6, 26.3) | 17.5 (11.4, 27.3) | 17.2 (13.7, 25.4) | 0.63 |
| Urea, mg/dL | 37.2 (26.9, 56.2) | 37.5 (24.5, 58.4) | 36.8 (29.3, 54.4) | 0.69 |
| Creatinine, mg/dL | 0.64 (0.51, 0.94) | 0.62 (0.49, 0.85) | 0.70 (0.55, 1.23) | 0.10 |
| Sodium, mmol/L | 137 (135, 138) | 137 (134, 138) | 137 (135, 138) | 0.97 |
| Potassium, mmol/L | 4.20 (3.80, 4.48) | 4.19 (3.78, 4.50) | 4.20 (3.78, 4.42) | 0.73 |
| Chlorine, mmol/L | 104 (101, 107) | 104 (102, 107) | 105 (100, 107) | 0.85 |
| Calcium, mg/dL | 8.80 (8.35, 9.36) | 8.70 (7.73, 9.39) | 8.84 (8.40, 9.34) | 0.65 |
| Phosphorus, mg/dL | 3.69 (3.11, 4.38) | 3.51 (3.00, 4.29) | 3.93 (3.26, 4.62) | 0.41 |
| Magnesium, mg/dL | 1.90 (1.71, 2.02) | 1.92 (1.70, 2.05) | 1.90 (1.72, 2.00) | 0.42 |
| C-reactive protein, mg/dL | 2.18 (0.67, 4.72) | 2.38 (0.51, 8.41) | 2.15 (0.79, 4.26) | 0.85 |
| Triglycerides, mg/dL | 124 (98.5, 194) | 118 (99.0, 175) | 144 (79.0, 237) | 0.51 |

Data are presented as median (25^th^, 75^th^ percentile). Statistical analysis was performed using Mann-Whitney U test. ALT: alanine aminotransferase; AST: aspartate aminotransferase; BUN: blood urea nitrogen; CLABSI: Central Line-Associated Bloodstream Infection.
