# Supplementary material for: Incidence and factors associated with central line-associated bloodstream infection in patients with chronic intestinal failure. A 20-year retrolective cohort
Source: PLoS One. 2026 Jan 6;21(1):e0340064. doi: 10.1371/journal.pone.0340064 (PMC12774362; doi:10.1371/journal.pone.0340064)
Supplement: S2 Table — (DOCX) [file pone.0340064.s003.docx]

**Table S3. Poisson regression analysis of variables associated with the incidence of CLABSI.**

| Variable | IR | 95% CI | Deviance/df value | AIC | P- value |
| --- | --- | --- | --- | --- | --- |
| Sepsis | 1.12 | 0.78-1.60 | 0.73 | 100.7 | 0.51 |
| Thrombosis | 1.07 | 0.73-1.57 | 0.74 | 100.9 | 0.71 |
| Additional use of CVC | 1.11 | 0.75-1.66 | 0.73 | 100.7 | 0.58 |
| Depression | 1.43 | 1.04-1.97 | 0.66 | 96.5 | **0.028** |
| Decompressive gastrostomy | 1.01 | 0.64-1.56 | 0.73 | 101.1 | 0.98 |
| Duration of HPN | 1.00 | 0.99-1.00 | 0.73 | 100.3 | 0.44 |
| Age | 0.99 | 0.98-1.00 | 0.72 | 99.8 | 0.24 |
| Sex (male) | 0.93 | 0.66-1.31 | 0.74 | 100.9 | 0.71 |
| Tobacco | 0.82 | 0.59-1.14 | 0.72 | 99.8 | 0.25 |
| Alcohol | 1.01 | 0.38-2.67 | 0.74 | 101.1 | 0.98 |
| Drugs | 1.25 | 0.49-3.21 | 0.73 | 100.8 | 0.63 |
| Presence of a stoma | 1.30 | 0.94-1.80 | 0.70 | 98.7 | 0.11 |
| Enteral access | 0.78 | 0.56-1.09 | 0.71 | 99.6 | 0.15 |
| Diabetes | 1.53 | 0.72-3.24 | 0.71 | 99.3 | 0.26 |
| Hypertension | 1.14 | 0.63-2.06 | 0.73 | 101.0 | 0.64 |
| Chronic kidney disease | 1.19 | 0.71-1.99 | 0.73 | 100.5 | 0.49 |
| Pneumonia | 1.11 | 0.75-1.66 | 0.73 | 100.7 | 0.58 |
| Cancer | 0.82 | 0.58-1.14 | 0.72 | 99.9 | 0.25 |
| Number of comorbidities |  |  | 0.70 | 100.2 |  |
| 1-3 | 1 |  |  |  |  |
| 4-6 | 1.30 | 0.90-1.87 |  |  | 0.15 |
| >6 | 1.42 | 0.95-2.14 |  |  | 0.08 |
| Weight | 0.99 | 0.98-1.00 | 0.73 | 100.7 | 0.55 |
| BMI | 0.96 | 0.88-1.04 | 0.73 | 101.2 | 0.38 |
| Parenteral nutrition | 0.77 | 0.55-1.01 | 0.71 | 99.5 | 0.15 |
| Hydration | 1.06 | 0.68-1.65 | 0.74 | 101.0 | 0.78 |
| Oral intake | 1.26 | 0.84-1.86 | 0.72 | 100.1 | 0.25 |
| Type of central venous access |  |  | 0.71 | 100.5 |  |
| Standard | 1 |  |  |  |  |
| Implanted port | 1.01 | 0.67-1.54 |  |  | 0.92 |
| Hickman | 1.35 | 0.89-2.05 |  |  | 0.14 |
| Site of insertion |  |  | 0.72 | 102.4 |  |
| Right Jugular | 1 |  |  |  |  |
| Left Jugular | 1.34 | 0.52-3.44 |  |  | 0.53 |
| Right Subclavia | 1.39 | 0.80-2.40 |  |  | 0.23 |
| Left Subclavia | 1.87 | 0.26-13.4 |  |  | 0.53 |
| Tunneled | 1.09 | 0.68-1.73 | 0.74 | 100.9 | 0.71 |
| Daily frequency of infusion | 1.02 | 0.70-1.50 | 0.74 | 101.0 | 0.88 |
| C-reactive protein | 0.93 | 0.77-1.11 | 0.66 | 55.3 | 0.42 |

CLABSI: Central Line-Associated Bloodstream Infection. CVC: central venous catheter; HPN: home parenteral nutrition; BMI: body mass index. AIC: Akaike information criterion; df: degree of freedom. Statistical analysis was performed with Poisson regression.
