# Supplementary material for: Incidence and factors associated with central line-associated bloodstream infection in patients with chronic intestinal failure. A 20-year retrolective cohort
Source: PLoS One. 2026 Jan 6;21(1):e0340064. doi: 10.1371/journal.pone.0340064 (PMC12774362; doi:10.1371/journal.pone.0340064)
Supplement: S3 Table — (DOCX) [file pone.0340064.s004.docx]

**Table S4. Multivariate Poisson regression analysis of variables associated with the incidence of CLABSI**

| Variable | IR | 95% CI | Deviance/df value | AIC | P- value |
| --- | --- | --- | --- | --- | --- |
| Sepsis | 1.32 | 0.61-2.85 | 0.73 | 104.6 | 0.48 |
| Thrombosis | 1.05 | 0.46-2.38 | 0.74 | 105.0 | 0.89 |
| Additional use of CVC | 1.21 | 0.53-2.75 | 0.74 | 104.8 | 0.63 |
| Depression | 2.38 | 0.93-6.04 | 0.67 | 101.3 | **0.06** |
| Decompressive gastrostomy | 0.95 | 0.35-2.58 | 0.74 | 105.0 | 0.93 |
| Duration of HPN | 1.00 | 1.00-1.00 | 0.74 | 104.6 | 0.48 |
| Tobacco | 0.59 | 0.24-1.45 | 0.72 | 103.7 | 0.25 |
| Alcohol | 1.08 | 0.39-3.01 | 0.74 | 105.0 | 0.87 |
| Drugs | 1.13 | 0.24-5.25 | 0.74 | 105.0 | 0.87 |
| Presence of a stoma | 1.91 | 0.80-4.54 | 0.70 | 102.7 | 0.14 |
| Enteral access | 0.52 | 0.17-1.55 | 0.71 | 103.5 | 0.24 |
| Diabetes | 2.24 | 0.78-6.38 | 0.71 | 103.0 | 0.13 |
| Hypertension | 2.62 | 0.68-10.0 | 0.71 | 103.2 | 0.16 |
| Chronic kidney disease | 1.96 | 0.71-5.41 | 0.72 | 103.5 | 0.19 |
| Pneumonia | 1.12 | 0.48-2.61 | 0.74 | 105.0 | 0.78 |
| Cancer | 0.67 | 0.26-1.68 | 0.73 | 104.3 | 0.39 |
| Number of comorbidities |  |  | 0.70 | 103.9 |  |
| 1-3 | 1 |  |  |  |  |
| 4-6 | 1.96 | 0.51-7.54 |  |  | 0.32 |
| >6 | 2.87 | 0.80-10.2 |  |  | 0.10 |
| Parenteral nutrition | 0.48 | 0.14-1.62 | 0.71 | 103.4 | 0.23 |
| Hydration | 1.36 | 0.50-3.67 | 0.74 | 104.7 | 0.53 |
| Oral intake | 1.77 | 0.41-7.55 | 0.73 | 104.3 | 0.43 |
| Type of central venous access |  |  | 0.71 | 100.5 |  |
| Standard | 1 |  | 0.71 | 104.7 |  |
| Implanted port | 1.30 | 0.38-4.38 |  |  | 0.67 |
| Hickman | 1.89 | 0.82-4.36 |  |  | 0.13 |
| Site of insertion |  |  | 0.73 | 107.0 |  |
| Right Jugular | 1 |  |  |  |  |
| Left Jugular | 1.95 | 0.43-8.75 |  |  | 0.38 |
| Right Subclavia | 1.68 | 0.66-4.30 |  |  | 0.27 |
| Left Subclavia | 2.52 | 0.32-19.7 |  |  | 0.37 |
| Tunneled | 1.28 | 0.38-4.33 | 0.74 | 104.9 | 0.68 |
| Daily frequency of infusion | 0.98 | 0.42-2.27 | 0.74 | 105.0 | 0.96 |
| C-reactive protein | 0.94 | 0.75-1.18 | 0.71 | 61.8 | 0.96 |

CLABSI: Central Line-Associated Bloodstream Infection. CVC: central venous catheter; HPN: home parenteral nutrition; BMI: body mass index. AIC: Akaike information criterion; df: degree of freedom. Statistical analysis was performed with Poisson regression. *The p-value was adjusted by sex, BMI and age variables.
