# Supplementary material for: Incidence and factors associated with central line-associated bloodstream infection in patients with chronic intestinal failure. A 20-year retrolective cohort
Source: PLoS One. 2026 Jan 6;21(1):e0340064. doi: 10.1371/journal.pone.0340064 (PMC12774362; doi:10.1371/journal.pone.0340064)
Supplement: S4 Table — (DOCX) [file pone.0340064.s005.docx]

**Table S5. Poisson regression analysis of variables associated with the incidence of polymicrobial CLABSI.**

| Variable | IR | 95% CI | P- value |  |
| --- | --- | --- | --- | --- |
| Age | 0.99 | 0.96-1.02 | 0.75 |  |
| Duration of OIS | 1.00 | 0.99-1.00 | 0.69 |  |
| Sex (male) | 1.50 | 0.49-4.58 | 0.47 |  |
| Tobacco | 2.85 | 0.95-8.48 | **0.06** |  |
| Alcohol | 1.52 | 0.46-4.94 | 0.48 |  |
| Sepsis | 1.20 | 0.15-9.29 | 0.85 |  |
| Thrombosis | 1.18 | 0.39-3.53 | 0.75 |  |
| Additional use of CVC | 0.44 | 0.09-2.00 | 0.29 |  |
| Depression | 0.65 | 0.21-2.00 | 0.45 |  |
| Decompressive gastrostomy | 1.27 | 0.39-4.14 | 0.68 |  |
| Presence of a stoma | 0.88 | 0.28-2.69 | 0.82 |  |
| Enteral access | 1.27 | 0.39-4.14 | 0.68 |  |
| Diabetes | 2.31 | 0.71-7.50 | 0.16 |  |
| Hypertension | 1.22 | 0.27-5.53 | 0.79 |  |
| Chronic kidney disease | 0.34 | 0.04-2.67 | 0.30 |  |
| Pneumonia | 1.31 | 0.42-4.01 | 0.63 |  |
| Cancer | 0.73 | 0.20-2.66 | 0.73 |  |
| Number of comorbidities  1-3  4-6  >7 | 1  0.61  0.38 | 0.15-2.46  0.08-1.70 | 0.49  0.20 |  |
| Weight | 1.0 | 0.96-1.03 | 0.97 |  |
| BMI | 1.01 | 0.89-1.14 | 0.85 |  |
| Parenteral nutrition | 0.65 | 0.20-2.13 | 0.48 |  |
| Hydration  Both  Oral intake | 1.07  0.81 | 0.18-3.67  0.33-3.47 | 0.90  0.79 |  |
| Type of CVA  Standard  Implanted port  Hickman | 1  0.27  0.54 | 0.03-2.16  0.16-1.79 | 0.21  0.31 | |
| Site of insertion  Right Jugular  Left Jugular  Right Subclavian  Left Subclavian | 1  3.33  1.66  3.33 | 0.67-16.5  0.47-5.90  0.40-27.6 | 0.14  0.42  0.26 | |
| Tunneled | 1.28 | 0.16-9.88 | 0.80 | |
| Daily frequency of infusion | 1.15 | 0.31-4.21 | 0.82 | |
| C-reactive protein | 0.93 | 0.68-1.26 | 0.64 | |

CLABSI: Central Line-Associated Bloodstream Infection. CVC: central venous catheter; CVA: central venous access; OIS: Outpatient intravenous supplementation; BMI: body mass index. Statistical analysis was performed with Poisson regression.
